# Biphenyl-3,3′,4,4′-tetra­amine

**DOI:** 10.1107/S1600536810012511

**Published:** 2010-04-14

**Authors:** Hui-Fen Qian, Wei Huang

**Affiliations:** a College of Sciences, Nanjing University of Technology, Nanjing 210009, People’s Republic of China; b State Key Laboratory of Coordination Chemistry, Nanjing National Laboratory of Microstructures, School of Chemistry and Chemical Engineering, Nanjing University, Nanjing 210093, People’s Republic of China

## Abstract

The title compound, C_12_H_14_N_4_, has a crystallographically imposed centre of symmetry. Inter­molecular N—H⋯N hydrogen bonds between amino groups link adjacent mol­ecules into a three-dimensional network where ten-membered hydrogen-bonded rings are observed.

## Related literature

For a related compound, see: Dobrzycki & Wozniak (2007 ▶).
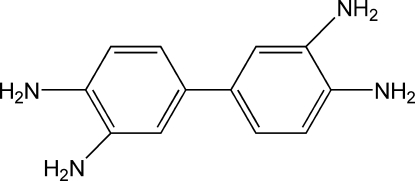


## Experimental

### 

#### Crystal data

C_12_H_14_N_4_
*M* *_r_* = 214.27
Monoclinic, 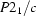

*a* = 9.646 (4) Å
*b* = 7.476 (3) Å
*c* = 7.751 (3) Å
β = 95.773 (5)°
*V* = 556.1 (4) Å^3^
*Z* = 2
Mo *K*α radiation
μ = 0.08 mm^−1^
*T* = 291 K
0.14 × 0.12 × 0.10 mm

#### Data collection

Bruker SMART 1K CCD area-detector diffractometer
Absorption correction: multi-scan (*SADABS*; Bruker, 2000 ▶) *T*_min_ = 0.989, *T*_max_ = 0.992
2698 measured reflections
979 independent reflections
724 reflections with *I* > 2σ(*I*)
*R*_int_ = 0.075

#### Refinement

*R*[*F* ^2^ > 2σ(*F* ^2^)] = 0.051
*wR*(*F* ^2^) = 0.156
*S* = 1.09
979 reflections
73 parameters
H-atom parameters constrained
Δρ_max_ = 0.18 e Å^−3^
Δρ_min_ = −0.30 e Å^−3^

### 

Data collection: *SMART* (Bruker, 2000 ▶); cell refinement: *SMART*; data reduction: *SAINT* (Bruker, 2000 ▶); program(s) used to solve structure: *SHELXTL* (Sheldrick, 2008 ▶); program(s) used to refine structure: *SHELXTL*; molecular graphics: *SHELXTL*; software used to prepare material for publication: *SHELXTL*.

## Supplementary Material

Crystal structure: contains datablocks global, I. DOI: 10.1107/S1600536810012511/bv2140sup1.cif

Structure factors: contains datablocks I. DOI: 10.1107/S1600536810012511/bv2140Isup2.hkl

Additional supplementary materials:  crystallographic information; 3D view; checkCIF report

## Figures and Tables

**Table 1:** Hydrogen-bond geometry (Å, °)

*D*—H⋯*A*	*D*—H	H⋯*A*	*D*⋯*A*	*D*—H⋯*A*
N1—H1*A*⋯N2^i^	0.90	2.39	3.224 (2)	154
N2—H2*A*⋯N1^ii^	0.90	2.35	3.124 (2)	145

Symmetry codes: (i) 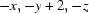; (ii) 
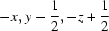
.

**Fig. 1.**
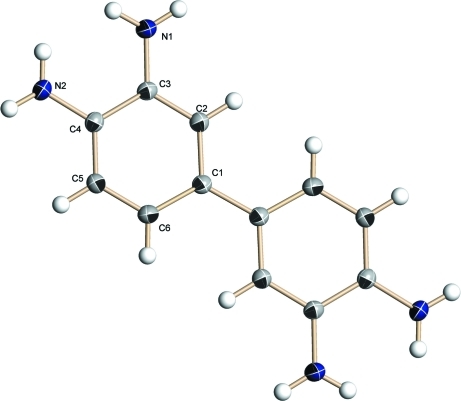
The molecular structure of the title compound with the atom-numbering scheme. Displacement ellipsoids are drawn at the 30% probability level.

**Fig. 2.**
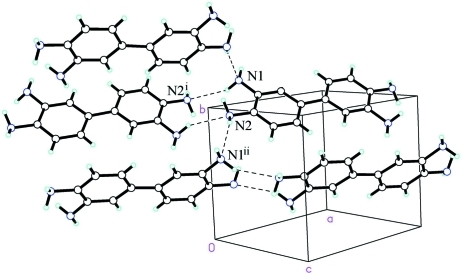
Perspective view of the hydrogen bonding interactions in the crystal packing of (I), where the hydrogen bonds are shown as dashed lines. [Symmetry codes: (i) -x, -y + 2, -z; (ii) -x, y - 1/2, -z + 1/2.]

## supplementary crystallographic information

### Comment

The crystal structure of 3,3',4,4'-tetrammoniobiphenyl tetrachloride dihydrate (Dobrzycki & Wozniak, 2007) has been reported in literature. In this paper, we report the X-ray single-crystal structure of 3,3',4,4'-tetrammoniobiphenyl (I).

The molecular structure of (I) is illustrated in Fig. 1. Two amino groups in the 3-position lie in the opposite sides of the molecular plane. The dihedral angle between phenyl rings of adjacent molecules is 86.3 (2)°. Intermolecular N—H···N hydrogen bonds between amino groups link adjacent molecules into a three-dimensional network, where ten-membered hydrogen-bonded rings are observed (Fig. 2).

### Experimental

The title compound was purchased directly from TCI. Single crystals suitable for X-ray diffraction were grown from a methanol solution by slow evaporation in air at room temperature for one week.

### Refinement

H atoms were placed in geometrically idealized positions and refined as riding, with C—H = 0.93 Å and N—H = 0.86–0.90 Å, and with *U*_iso_(H) = 1.2*U*_eq_(C,N).

### Crystal data

**Table:**

C_12_H_14_N_4_	*F*(000) = 228
*M**_r_* = 214.27	*D*_x_ = 1.280 Mg m^−^^3^
Monoclinic, *P*2_1_/*c*	Mo *K*α radiation, λ = 0.71073 Å
Hall symbol: -P 2ybc	Cell parameters from 931 reflections
*a* = 9.646 (4) Å	θ = 2.5–27.0°
*b* = 7.476 (3) Å	µ = 0.08 mm^−^^1^
*c* = 7.751 (3) Å	*T* = 291 K
β = 95.773 (5)°	Block, colourless
*V* = 556.1 (4) Å^3^	0.14 × 0.12 × 0.10 mm
*Z* = 2	

### Data collection

**Table:**

Bruker SMART 1K CCD area-detector diffractometer	979 independent reflections
Radiation source: fine-focus sealed tube	724 reflections with *I* > 2σ(*I*)
graphite	*R*_int_ = 0.075
ω scans	θ_max_ = 25.0°, θ_min_ = 2.1°
Absorption correction: multi-scan (*SADABS*; Bruker, 2000)	*h* = −9→11
*T*_min_ = 0.989, *T*_max_ = 0.992	*k* = −6→8
2698 measured reflections	*l* = −8→9

### Refinement

**Table:**

Refinement on *F*^2^	Primary atom site location: structure-invariant direct methods
Least-squares matrix: full	Secondary atom site location: difference Fourier map
*R*[*F*^2^ > 2σ(*F*^2^)] = 0.051	Hydrogen site location: inferred from neighbouring sites
*wR*(*F*^2^) = 0.156	H-atom parameters constrained
*S* = 1.09	*w* = 1/[σ^2^(*F*_o_^2^) + (0.0926*P*)^2^ + 0.0016*P*] where *P* = (*F*_o_^2^ + 2*F*_c_^2^)/3
979 reflections	(Δ/σ)_max_ < 0.001
73 parameters	Δρ_max_ = 0.18 e Å^−^^3^
0 restraints	Δρ_min_ = −0.30 e Å^−^^3^

### Special details

**Table:**

Experimental. The structure was solved by direct methods (Bruker, 2000) and successive difference Fourier syntheses.
Geometry. All e.s.d.'s (except the e.s.d. in the dihedral angle between two l.s. planes) are estimated using the full covariance matrix. The cell e.s.d.'s are taken into account individually in the estimation of e.s.d.'s in distances, angles and torsion angles; correlations between e.s.d.'s in cell parameters are only used when they are defined by crystal symmetry. An approximate (isotropic) treatment of cell e.s.d.'s is used for estimating e.s.d.'s involving l.s. planes.
Refinement. Refinement of *F*^2^ against ALL reflections. The weighted *R*-factor *wR* and goodness of fit *S* are based on *F*^2^, conventional *R*-factors *R* are based on *F*, with *F* set to zero for negative *F*^2^. The threshold expression of *F*^2^ > σ(*F*^2^) is used only for calculating *R*-factors(gt) *etc*. and is not relevant to the choice of reflections for refinement. *R*-factors based on *F*^2^ are statistically about twice as large as those based on *F*, and *R*- factors based on ALL data will be even larger.

### Fractional atomic coordinates and isotropic or equivalent isotropic displacement parameters (Å^2^)

**Table:**

	*x*	*y*	*z*	*U*_iso_*/*U*_eq_	
C1	0.42719 (17)	0.9895 (2)	0.4590 (2)	0.0335 (5)	
C2	0.37707 (18)	1.0872 (2)	0.3125 (2)	0.0356 (5)	
H2	0.4378	1.1639	0.2629	0.043*	
C3	0.24074 (18)	1.0749 (2)	0.2378 (2)	0.0336 (5)	
C4	0.14684 (18)	0.9615 (2)	0.3120 (2)	0.0341 (5)	
C5	0.1965 (2)	0.8586 (2)	0.4523 (2)	0.0391 (6)	
H5	0.1367	0.7785	0.4991	0.047*	
C6	0.3330 (2)	0.8714 (3)	0.5255 (2)	0.0421 (6)	
H6	0.3629	0.8003	0.6205	0.051*	
N1	0.18955 (16)	1.1838 (2)	0.0986 (2)	0.0442 (5)	
H1A	0.1515	1.1130	0.0127	0.053*	
H1B	0.2437	1.2600	0.0562	0.053*	
N2	0.00747 (15)	0.9522 (2)	0.23637 (19)	0.0418 (5)	
H2A	−0.0484	0.9167	0.3161	0.050*	
H2B	−0.0130	1.0651	0.2025	0.050*	

### Atomic displacement parameters (Å^2^)

**Table:**

	*U*^11^	*U*^22^	*U*^33^	*U*^12^	*U*^13^	*U*^23^
C1	0.0327 (11)	0.0338 (10)	0.0336 (10)	0.0017 (8)	0.0013 (8)	−0.0006 (8)
C2	0.0326 (11)	0.0381 (11)	0.0362 (10)	−0.0008 (8)	0.0043 (8)	0.0023 (8)
C3	0.0355 (11)	0.0348 (10)	0.0300 (9)	0.0026 (8)	0.0004 (8)	−0.0012 (7)
C4	0.0327 (11)	0.0353 (10)	0.0337 (10)	−0.0013 (8)	0.0007 (8)	−0.0053 (8)
C5	0.0376 (12)	0.0392 (11)	0.0397 (11)	−0.0082 (8)	−0.0003 (9)	0.0049 (8)
C6	0.0422 (12)	0.0420 (11)	0.0404 (11)	−0.0036 (9)	−0.0046 (9)	0.0092 (8)
N1	0.0434 (11)	0.0480 (10)	0.0396 (10)	−0.0045 (7)	−0.0033 (8)	0.0113 (7)
N2	0.0324 (10)	0.0493 (11)	0.0424 (10)	−0.0036 (7)	−0.0026 (7)	0.0017 (7)

### Geometric parameters (Å, °)

**Table:**

C1—C2	1.395 (3)	C4—N2	1.413 (2)
C1—C6	1.401 (3)	C5—C6	1.384 (3)
C1—C1^i^	1.491 (3)	C5—H5	0.9300
C2—C3	1.386 (2)	C6—H6	0.9300
C2—H2	0.9300	N1—H1A	0.8999
C3—N1	1.401 (2)	N1—H1B	0.8600
C3—C4	1.405 (2)	N2—H2A	0.9000
C4—C5	1.379 (3)	N2—H2B	0.9000
			
C2—C1—C6	116.41 (17)	C4—C5—C6	121.72 (17)
C2—C1—C1^i^	121.8 (2)	C4—C5—H5	119.1
C6—C1—C1^i^	121.8 (2)	C6—C5—H5	119.1
C3—C2—C1	122.83 (17)	C5—C6—C1	121.21 (18)
C3—C2—H2	118.6	C5—C6—H6	119.4
C1—C2—H2	118.6	C1—C6—H6	119.4
C2—C3—N1	121.97 (16)	C3—N1—H1A	108.3
C2—C3—C4	119.50 (16)	C3—N1—H1B	119.9
N1—C3—C4	118.29 (16)	H1A—N1—H1B	108.9
C5—C4—C3	118.20 (17)	C4—N2—H2A	109.9
C5—C4—N2	122.70 (16)	C4—N2—H2B	104.2
C3—C4—N2	119.05 (16)	H2A—N2—H2B	110.4
			
C6—C1—C2—C3	2.1 (3)	N1—C3—C4—N2	4.4 (2)
C1^i^—C1—C2—C3	−177.55 (18)	C3—C4—C5—C6	3.2 (3)
C1—C2—C3—N1	175.14 (17)	N2—C4—C5—C6	−179.28 (17)
C1—C2—C3—C4	0.8 (3)	C4—C5—C6—C1	−0.3 (3)
C2—C3—C4—C5	−3.4 (3)	C2—C1—C6—C5	−2.3 (3)
N1—C3—C4—C5	−177.99 (15)	C1^i^—C1—C6—C5	177.30 (19)
C2—C3—C4—N2	178.99 (15)		

Symmetry codes: (i) −*x*+1, −*y*+2, −*z*+1.

### Hydrogen-bond geometry (Å, °)

**Table:**

*D*—H···*A*	*D*—H	H···*A*	*D*···*A*	*D*—H···*A*
N1—H1A···N2^ii^	0.90	2.39	3.224 (2)	154
N2—H2A···N1^iii^	0.90	2.35	3.124 (2)	145

Symmetry codes: (ii) −*x*, −*y*+2, −*z*; (iii) −*x*, *y*−1/2, −*z*+1/2.
